# Making a difference in multi-data-set crystallography: simple and deterministic data-scaling/selection methods

**DOI:** 10.1107/S2059798320006348

**Published:** 2020-06-17

**Authors:** Greta M. Assmann, Meitian Wang, Kay Diederichs

**Affiliations:** a Department of Biology, University of Konstanz, Box 647, D-78457 Konstanz, Germany; b Swiss Light Source, Paul Scherrer Institute, CH-5232 Villigen, Switzerland

**Keywords:** serial crystallography, non-isomorphism, data selection, data scaling, SAD phasing

## Abstract

Phasing by single-wavelength anomalous diffraction (SAD) from multiple crystallographic data sets can be particularly demanding because of the weak anomalous signal and possible non-isomorphism. The identification and exclusion of non-isomorphous data sets by suitable indicators is therefore indispensable. Here, simple and robust data-selection methods are described. A multi-dimensional scaling procedure is first used to identify data sets with large non-isomorphism relative to clusters of other data sets. Within each cluster that it identifies, further selection is based on the weighted ΔCC_1/2_, a quantity representing the influence of a set of reflections on the overall CC_1/2_ of the merged data. The anomalous signal is further improved by optimizing the scaling protocol. The success of iterating the selection and scaling steps was verified by substructure determination and subsequent structure solution. Three serial synchrotron crystallography (SSX) SAD test cases with hundreds of partial data sets and one test case with 62 complete data sets were analyzed. Structure solution was dramatically simplified with this procedure, and enabled solution of the structures after a few selection/scaling iterations. To explore the limits, the procedure was tested with much fewer data than originally required and could still solve the structure in several cases. In addition, an SSX data challenge, minimizing the number of (simulated) data sets necessary to solve the structure, was significantly underbid.

## Abstract 2

Fast and deterministic methods, based on multi-dimensional scaling and weighted ΔCC_1/2_, to reject non-isomorphous data sets in multi-data-set crystallography are described, and their successful application to several difficult projects where phasing is based on weak anomalous signal is reported.

## 1. Introduction   

Obtaining large crystals and solving the phase problem remain the major bottlenecks in macromolecular crystallography. To overcome the problem of a lack of sufficiently large crystals for collecting a complete data set with little radiation damage, multi-crystal data-collection strategies were established early on and have recently experienced a renaissance (Kendrew *et al.*, 1960 ▸; Dickerson *et al.*, 1961 ▸; Ji *et al.*, 2010 ▸; Liu *et al.*, 2012 ▸; Akey *et al.*, 2014 ▸; Huang *et al.*, 2018 ▸). Serial synchrotron crystallography (SSX; Rossmann, 2014 ▸) typically collects a few degrees of rotation data from each of the small crystals available to the experimenter.

The term ‘SSX’ has recently been used in a wider sense, referring to fixed-target or injection-based single zero-rotation diffraction patterns (stills) from crystals exposed to monochromatic (Nogly *et al.*, 2015 ▸; Botha *et al.*, 2015 ▸; Owen *et al.*, 2017 ▸) or polychromatic (pink) radiation (Meents *et al.*, 2017 ▸; Martin-Garcia *et al.*, 2019 ▸). Serial femtosecond crystallo­graphy (SFX) takes this method to the extreme; it collects stills from numerous small crystals before destroying them using X-ray pulses generated by a free-electron laser.

If crystals are not rotated during exposure, monochromatic data sets contain fewer reflections than those from SSX with rotated crystals and all reflections are partials (Boutet *et al.*, 2012 ▸; Chapman *et al.*, 2011 ▸). Both methods ideally result in a complete data set if enough partial data sets are combined.

To overcome the phase problem, several strategies have been established and multiple-wavelength or single-wavelength anomalous diffraction (MAD or SAD) predominate in *de novo* structure determination (Hendrickson, 2014 ▸). Heavy-atom derivatization or selenomethionine substitution in proteins ensures the production of strong anomalous diffraction; however, even light native elements such as sulfur (*Z* = 16) in cysteine, and methionine and phosphorus (*Z* = 15) in nucleic acids suffice for the generation of a weak anomalous signal at low energies (Hendrickson & Teeter, 1981 ▸; Liu *et al.*, 2012 ▸). The expected anomalous signal relative to the normal signal can be estimated based on the composition of the sample, and the wavelength. For SAD the anomalous signal (Bijvoet diffraction ratio) typically varies between 1% and 5% of the total scattering signal (Watanabe *et al.*, 2005 ▸; Liu *et al.*, 2012 ▸), which is often weaker than the measurement error of an intensity value (Hendrickson, 1991 ▸). Therefore, high multiplicity is usually required. The combination of SAD and multi-crystal data-collection strategies could exacerbate the correct determination of the anomalous differences, as the weak anomalous signals of all data sets are required to be consistent (isomorphous) with each other.

Isomorphism of crystals in the literal sense denotes the conservation of morphology, which entails space group and unit-cell parameters. For crystallographic data sets, this concept extends to the diffracted intensities and the resulting models. Isomorphous data sets (crystals) thus represent the same atomic model; in the strict sense, they only differ randomly from each other, for example, owing to variation in the intensities resulting from the Poisson statistics of photon counting, and can be scaled and averaged (merged). On the other hand, non-isomorphous data sets (crystals) either represent different atomic models or crystal packings, or are affected by experimental deficiencies; their intensities differ both randomly and systematically and thus should not be averaged. A robust method to identify non-isomorphous data sets (crystals) is therefore crucial for SAD multi-crystal data collection and the accurate determination of atomic models.

Outlier data sets can potentially be identified by hierarchical cluster analysis (HCA), using deviations of their unit-cell parameters as a proxy for systematic differences (Foadi *et al.*, 2013 ▸). However, the similarity of unit-cell parameters is a necessary but not sufficient condition and the actual similarity of the diffraction is not assessed in the selection process, which therefore only identifies strongly deviating data sets (crystals). For SSX with partial data sets, the unit-cell-based method could further suffer from the unavoidable inaccuracy in the determination of the unit-cell parameters. HCA has also been employed based on the pairwise comparison of intensities of common reflections (Giordano *et al.*, 2012 ▸). Alternatively, the pairwise correlation of every single data set and the reference data set from all merged data sets has been used to reject data based on a chosen correlation cutoff (Huang *et al.*, 2018 ▸). The selection is based on correlation coefficients between intensities, but since a low correlation results from both non-isomorphism and weak exposure, the disadvantage is that weak (high random error) but isomorphous (low systematic error) data sets are rejected, which trades accuracy (correctness) for precision (internal consistency). Automated pipelines such as *MeshAndCollect* (Zander *et al.*, 2015 ▸) and *ccCluster* (Santoni *et al.*, 2017 ▸) with both unit-cell-based and intensity-based HCA selection have recently been established. Basu *et al.* (2019 ▸) provide another automated SSX software suite with selection of data based on unit-cell parameters, asymptotic *I*/σ (ISa) (Diederichs, 2010 ▸; Diederichs & Wang, 2017 ▸) or pairwise correlation coefficients. Another approach utilizes a genetic algorithm (Zander *et al.*, 2016 ▸; Foos *et al.*, 2019 ▸) that generates random combinations of data sets into subsets. These are then optimized according to an iteratively optimized fitness score derived from a weighted combination of *R*_meas_, 〈*I*/σ〉, CC_1/2_ (Karplus & Diederichs, 2012 ▸), complete­ness, multiplicity and, in the case of Foos *et al.* (2019 ▸), anomalous CC_1/2_ (called CC_anom overall_ by Foos and coworkers and termed CC_1/2_ano_ in this paper). This approach again optimizes precision but not necessarily accuracy, and may not scale well with increasing numbers of data sets.

For experimental phasing, some selection methods focus on the anomalous signal by calculating anomalous correlations and rejecting data sets with an (arbitrarily) ‘low’ anomalous correlation or ‘high’ *R*_merge_ (Akey *et al.*, 2014 ▸). The anomalous correlation between a single data set and a reference data set of all merged data sets, the relative anomalous correlation coefficient (RACC), was employed by Liu *et al.* (2012 ▸) and was further combined with cluster analysis dependent on both unit-cell parameters and intensity correlations. Yet another selection procedure combines frame rejection based on relative correlation coefficients (RCC) and CC_1/2_, crystal rejection based on Sm*R*_merge_ (smoothed-frame *R*_merge_, as reported in *AIMLESS*; Evans & Murshudov, 2013 ▸) and further subset selection based on anomalous correlation coefficients (ACCs; Guo *et al.*, 2018 ▸, 2019 ▸). As the existence of a Bijvoet partner in the data set is required for the calculation of an anomalous difference of a reflection, few (if any) reflections per data set are included in the calculation if the data sets are partial. The low number of reflections used, in combination with the weakness of the anomalous signal, dramatically decreases the significance of the calculated anomalous correlations. This effect is amplified the narrower the rotation range of the single data sets and the lower the symmetry of the space group, and therefore selection based on anomalous correlations may not always be feasible.

Brehm & Diederichs (2014 ▸) and Diederichs (2017 ▸) suggested a multi-dimensional scaling method for mapping differences between data sets to a low-dimensional space based on pairwise correlation coefficients. In this method, every data set is represented by a vector in a unit sphere; the angle between two vectors corresponds to their systematic difference, whereas the lengths of the vectors are related to the amount of random differences between the data sets. The identification of single data sets or data-set clusters showing systematic differences (non-isomorphism) can be performed, for example, by visual inspection or by cluster analysis of the low-dimensional arrangement of vectors representing the data sets. This method has since been used to remove the indexing ambiguity that exists in several point groups and also for specific combinations of unit-cell parameters when analyzing data sets in SSX or SFX (Brehm & Diederichs, 2014 ▸).

Following previous work (Karplus & Diederichs, 2012 ▸; Diederichs & Karplus, 2013 ▸; Assmann *et al.*, 2016 ▸), in this study we chose the numerical value of CC_1/2_ as an optimization target depending on the data sets included in scaling and merging. CC_1/2_ is a precision indicator for the scaled and merged data set which was originally based on the random assignment of observations to half-data sets. It allows the calculation of CC* which, in the absence of systematic errors, describes the correlation of the resulting data with the underlying ‘true’ signal. CC* (and thus CC_1/2_) provides a statistically valid guide to assess when data quality is limiting model improvement (Karplus & Diederichs, 2012 ▸). Assmann *et al.* (2016 ▸) suggested a method to detect data sets in a multi-crystal experiment that would result in a decrease of overall data quality, as assessed by CC_1/2_, if not rejected from data scaling and merging. A formula to calculate CC_1/2_ without random assignment was derived, which results in more precise values of CC_1/2_. This allowed the introduction of the ΔCC_1/2_ method for the identification of non-isomorphous data sets.

In this study, a combination and extension of the two methods (Diederichs, 2017 ▸; Assmann *et al.*, 2016 ▸) is proposed and analyzed using projects featuring multiple data sets obtained by the rotation method. The multi-dimensional scaling approach and the subsequent visualization of the low-dimensional space solution provides an initial tool to detect indexing ambiguities and data sets which display strong systematic differences. In a second step, optimization of the isomorphous or anomalous signal (CC_1/2_ or CC_1/2_ano_) by the iterative rejection of the data sets with the lowest ΔCC_1/2_ makes the key difference and allows simplified structure solution in challenging SAD test cases (data from Huang *et al.*, 2018 ▸; Akey *et al.*, 2014 ▸).

## 2. Methods and theory   

### 2.1. Processing and scaling of data sets   

All data sets were processed with *XDS* (Kabsch, 2010*a* ▸), and scaled with *XSCALE* (Kabsch, 2010*b* ▸). Since the standard deviations σ_*i*_ of the reflection intensities *I_i_* are used as weights *w_i_* = 1/σ^2^_*i*_ in scaling and merging, the error model of each data set, which serves to adjust the σ_*i*_ such that they match the observed differences between symmetry-related reflections, plays an important role. The *INTEGRATE* step of *XDS* derives a first estimate σ_0,*i*_ of σ_*i*_ from counting statistics, and inflates it to σ_*i*_ = 2(σ^2^_0,*i*_ + 0.0001*I_i_* ^2^)^1/2^, thus limiting the *I_i_*/σ_*i*_ values to at most 50. The error model is then adjusted in the *CORRECT* step of *XDS*. However, in the SSX case only few (or no) symmetry-related reflections per data set exist and the adjustment of the error model in *XDS* may be poorly determined or cannot be performed at all. This may lead to a biased weighting of data sets in the scaling procedure, and should be avoided. Consequently, we obtained the best results (see Section 3.4) when we prevented *XDS* from scaling and further adjusting the error model in its *CORRECT* step by using MINIMUM_I/SIGMA=50 in versions of *XDS* before October 2019 (and SNRC=50 thereafter), and thus postponed the scaling and calculation of the error model to *XSCALE*. However, this required the availability of the unscaled INTEGRATE.HKL reflection files. Some data sets were only available to us as XDS_ASCII.HKL files, the internal scale factors and error model of which had already been adjusted in *CORRECT* if there were symmetry-related reflections within the same data set. As we preferred to have *XSCALE* determine the scale and error model of each data set in the context of all other data sets, we wrote a small helper program *RESET_VARIANCE_MODEL* to (approximately) revert the adjustment of the error model, based on the two parameters of the error model as stored in the reflection file produced by *CORRECT*.

### 2.2. *XSCALE_ISOCLUSTER*   

Data sets can differ in as many ways as there are reflections. After merging and averaging symmetry-related reflections, a data set can therefore be represented as a point in a space that has as many dimensions as there are unique reflections. Since it is cumbersome to analyze data in high-dimensional space, we use dimensionality reduction to characterize and classify data sets in a low-dimensional space. To this end, Diederichs (2017 ▸) suggested a multi-dimensional scaling analysis that separates single data sets according to their random and systematic differences. Data sets are represented by vectors in low-dimensional space; this space has the shape of a unit sphere.

Numerically, the arrangement of vectors in low-dimensional space is obtained by minimization of the function Φ(**x**),
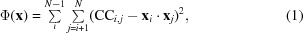
dependent on the differences of the pairwise correlation coefficients CC_*i*,*j*_ of data sets *i* and *j*, calculated from the intensities of common unique reflections, and the respective dot products of vectors **x**_*i*_, **x** *_j_* representing the data sets in low-dimensional space. At the minimum of the function, the dot products between any pair of vectors reproduce, in a least-squares sense, the correlation coefficients between the data sets that these vectors represent.

It has been shown (Diederichs, 2017 ▸) that the lengths of the vectors can be interpreted as the quantity CC* (Karplus & Diederichs, 2012 ▸), giving the correlation between the intensities of a data set and the true values. Moreover, the lengths of the vectors are inversely related to the amount of random error in the data sets, whereas their differences in direction represent their systematic differences. Data sets with vectors pointing in the same direction thus only differ in random error; if the vectors have the same length then the data sets also contain similar amounts of random errors. Short vectors represent noisy data sets; long vectors represent data sets with high signal-to-noise ratios and low random deviation from the ‘true’ data set, which would be located in the same direction but at a length of 1, *i.e.* on the surface of the sphere.

This method was implemented in the program *XSCALE_ISOCLUSTER*. The program reads the *XSCALE* output file (scaled but unmerged intensities) provided by the user and calculates pairwise correlation coefficients between data sets from averaged (within each data set) intensities of common reflections. Next, the solution vectors are constructed from the correlation coefficient matrix. The program writes a new XSCALE.INP file, which also reports, for each data set, the length of its vector and the angle with respect to the centre of gravity of all data sets. Additionally, a pseudo-PDB file with vector coordinates for visualization of the mutual arrangement of data sets is written. For this study, the program was run with the settings -nbin=1 (one resolution bin) and -dim=3 (representation in three dimensions).

### 2.3. The σ–τ method and calculation of ΔCC_1/2_: *XDSCC*12   

For the calculation of CC_1/2_, the observations of all experimental data sets are randomly assigned to two (ideally equally sized) half-data sets, and every unique reflection is merged individually within each half-data set (Karplus & Diederichs, 2012 ▸). In a previous study (Assmann *et al.*, 2016 ▸) another way to calculate CC_1/2_ was introduced to avoid the random assignment to the half-data sets. The calculation of CC_1/2_ is based on the Supplementary Material to Karplus & Diederichs (2012 ▸) and on Assmann *et al.* (2016 ▸),
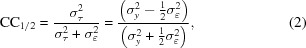
where σ_*y*_^2^ is the variance of the average intensities across the unique reflections of a resolution shell and ½σ_*∊*_^2^ is the average variance of the mean of the observations contributing to them. σ_τ_^2^, the variance of τ, is related to σ_*y*_^2^ by σ_*y*_^2^ = σ_τ_^2^ + ½σ_*∊*_^2^. For this study, we implemented the weighting of the intensities in the CC_1/2_ calculations in our program *XDSCC*12, which reads the reflection output file from *XSCALE* containing the scaled and unmerged intensities of all data sets.

We estimate σ_*∊*_^2^ from the unbiased weighted sample variance of the mean *s* ^2^_∊*w*_ (equations 4.22 and 4.23 in Bevington & Robinson, 2003 ▸) for a half-data set and use the standard deviations of the observations, modified by the error model determined for every partial data set by *XSCALE*, as weights. For each reflection *i* with observations *j*, the contribution 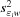 to *s* ^2^_∊*w*_ is calculated from the *n_i_* different data sets that include this particular reflection. Accounting for the reduced size of the half-data set requires division of 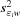 by *n_i_*/2 instead of *n_i_*, 
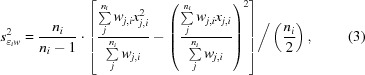
where *w*_*j*,*i*_ = 1/σ_*j*,*i*_^2^. We changed the calculation of frequency-weighted 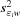 (3) to use reliability weights (following the notation used in Wikipedia; https://en.wikipedia.org/wiki/Weighted_arithmetic_mean#Reliability_weights), replacing *n_i_*/(*n_i_* − 1) with 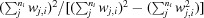 and *n*_*i*_/2 with 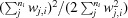, which resulted in
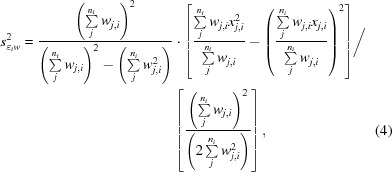
in which some terms cancel down. Finally, the variances 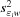 are averaged over all *N* unique reflections to obtain 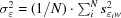.

The algorithm to optimize CC_1/2_ requires the calculation of CC_1/2,with-*i*_ for all of the data sets used and CC_1/2,without-*i*_, the CC_1/2_ for all data sets without the observations of one single data set *i*, for those unique reflections that are represented in *i* and excluding those that are only represented in *i*. Both CC_1/2,with-*i*_ and CC_1/2,without-*i*_ are calculated with the above formulas. The difference, given by
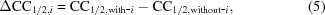
informs whether data set *i* improves (ΔCC_1/2,*i*_ > 0) or deterior­ates (ΔCC_1/2,*i*_ < 0) the merged data for the reflections represented in data set *i*. In our implementation, ΔCC_1/2,*i*_ is calculated for all resolution bins and averaged. To obtain more meaningful ΔCC_1/2_ differences that are independent of the magnitude of the CC values involved, the ΔCC_1/2_ values are by default modified by a Fisher transformation (Fisher, 1915 ▸), thus replacing (5) with
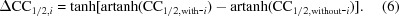
For example, this formula assigns the same value (about 0.01) to ΔCC_1/2_ if (CC_1/2,with-*i*_, CC_1/2,without-*i*_) is (0.0100, 0.0000), (0.2096, 0.2000), (0.9019, 0.9000) or (0.9902, 0.9900).

The equivalent quantities for the anomalous signal, CC_1/2_ano,with-*i*_, CC_1/2_ano,without-*i*_ and ΔCC_1/2_ano,*i*_, can be calculated analogously. Importantly, calculation of ΔCC_1/2_ano,*i*_ does not require both Bijvoet mates to be present in data set *i*.

ΔCC_1/2,*i*_ and ΔCC_1/2_ano,*i*_ values for each data set are reported by *XDSCC*12, and a file that may be edited and used as input to *XSCALE* is written out. This file is sorted by ΔCC_1/2,*i*_.

### 2.4. Iterative scaling and rejection   

We combined the calculation of a weighted and Fisher-transformed ΔCC_1/2_ with an iterative selection procedure. Firstly, all data sets (with σ values as obtained in *INTEGRATE*, *i.e.* without adjustment in *CORRECT*) are scaled with *XSCALE*. The following steps are then performed.

(i) *XDSCC*12 is run with the options -nbin and -dmax. We use -nbin 1 to maximize the number of common reflections per pairwise data-set combination. Using the -dmax option, a high-resolution cutoff is chosen such that only statistically significant data are included.
(ii) The newly generated XSCALE.INP file (written by *XDSCC*12) containing all data sets sorted by ΔCC_1/2_ is inspected and the worst data sets (at least one data set and at most 1% of the total number) are removed from it. Data sets with positive ΔCC_1/2,*i*_ should not be removed since this would impair the merged CC_1/2_. Sorting of the data sets by their anomalous contribution (ΔCC_1/2_ano,*i*_) is also possible, but is only recommended when complete data sets are used (see Section 3.6). Sorting by ΔCC_1/2_ also allows the best data set to be subsequently used as a reference data set (with a scale of 1 and a relative *B* factor of 0) in *XSCALE*, which is generally desirable in scaling multiple data sets.
(iii) A new scaling run with *XSCALE* is performed with the reduced number of data sets. The resulting reflection file can be used for structure-solution attempts.

Steps (i)–(iii) may be iterated as long as there remain data sets with significant negative ΔCC_1/2,*i*_. Because ΔCC_1/2_ has limited precision (it has a standard error inversely proportional to the square root of the number of reflections), data sets with ΔCC_1/2,*i*_ around 0 should not be rejected: these may just be weak, and rejection without good reason may ultimately reduce the completeness. Usually, the execution of a few rejection iterations is enough to improve data quality, and may enable structure solution.

### 2.5. Availability and use of software   

The *XSCALE_ISOCLUSTER* and *XDSCC*12 programs for Linux and MacOS are available from their respective XDSwiki articles (https://strucbio.biologie.uni-konstanz.de/xdswiki/index.php/Xscale_isocluster), which also document them. The programs have negligible runtime; they can be easily integrated into scripts and are therefore suitable for automation.

### 2.6. Projects and their data sets   

Three projects with partial experimental SSX data sets, one project with complete experimental SSX data sets and one project with simulated partial SSX data sets were examined in this study. Their statistics can be found in Table 1 ▸.

#### 2.6.1. Partial experimental SSX data sets: BacA, PepT and LspA   

Partial data sets were kindly provided by Huang *et al.* (2018 ▸) as individual XDS_ASCII.HKL files for all data sets of the three proteins BacA (El Ghachi *et al.*, 2018 ▸), PepT (Lyons *et al.*, 2014 ▸) and LspA (Vogeley *et al.*, 2016 ▸). The error model of every XDS_ASCII.HKL file was reset using *RESET_VARIANCE_MODEL*. The parameter MINIMUM_I/SIGMA=0, adopted from Huang *et al.* (2018 ▸), was used in *XSCALE* (or SNRC=0.1 in *XSCALE* built on or after 15 October 2019). The substructure was determined with *SHELXD* (version 2013/2; Sheldrick, 2010 ▸), with resolution cutoffs of 3.3, 3.5 and 4.2 Å for BacA, PepT and LspA, respectively, and NTRY 25000; phase improvement and extension as well as autotracing was performed with *SHELXE* (version 01/2019; Sheldrick, 2010 ▸) with the options -s0.60 (solvent fraction) -a25 (autotracing cycles) -q (α-helical search) -z (substructure optimization) for BacA, -s0.55 -a25 -q -z for PepT and -s0.65 -a25 -q -z for LspA or with the *CRANK*2 pipeline (Skubák & Pannu, 2013 ▸) for BacA and LspA.

#### 2.6.2. Complete experimental data sets: NS1   

Raw data for NS1 were kindly provided by Akey *et al.* (2014 ▸) and served as an example of complete SSX data. *XDS* processing with SNRC=50 from 28 crystals with on average two wedges each resulted in 62 complete data sets as XDS_ASCII.HKL files. Scaling and merging was performed with *XSCALE* and SNRC=0.1. The substructure was determined with *SHELXD* with a resolution cutoff of 4.2 Å; phase refinement, auto­tracing and refinement were performed with the *CRANK*2 pipeline starting from the previously found substructure.

#### 2.6.3. Simulated SSX data sets: modified 1g1c   

Artificial data sets were provided by Holton (2019 ▸). These are based on squared structure amplitudes calculated from the coordinates of PDB entry 1g1c (Mayans *et al.*, 2001 ▸), but with slightly changed unit-cell parameters and crystal packing. The artificial intensities were modified to simulate significant radiation damage. Additional systematic errors were introduced in the frame-simulation program *MLFSOM* (Holton *et al.*, 2014 ▸).

After processing the 100 simulated SSX data sets (three frames of 1° rotation each) with *XDS* (SNRC=50), indexing ambiguities were analyzed with *XSCALE_ISOCLUSTER*. Reindexing, scaling and merging were performed with *XSCALE*. The parameters NBATCH=3 CORRECTIONS=DECAY ABSORPTION were used. The substructure was determined with *SHELXD* with a resolution cutoff of 3.5 Å; phase refinement and autotracing was performed with *SHELXE* with the options -s0.53 (solvent fraction) -L1 (minimum chain length) -B3 (β-sheet search) -a100 (autotracing cycles) as suggested by Holton (2019 ▸).

### 2.7. Automatic model building and refinement   

CC_trace/nat_ > 25% was used as an indicator of successful structure solution (Thorn & Sheldrick, 2013 ▸). The structures of BacA, LspA and NS1 could not be solved with *SHELXE*; for these we used *CRANK*2 and monitored *R*_work_ and *R*_free_ from the *REFMAC* (Murshudov *et al.*, 2011 ▸) refinement which is reported by the last *CRANK*2 step. Refinements in the PepT project were performed with *phenix.refine* (Liebschner *et al.*, 2019 ▸) using PDB entry 4xnj as a model, after ‘shaking’ using the options sites.shake=0.5 and adp.set_b_iso=53.

### 2.8. Flowchart   

**Figure 1:**
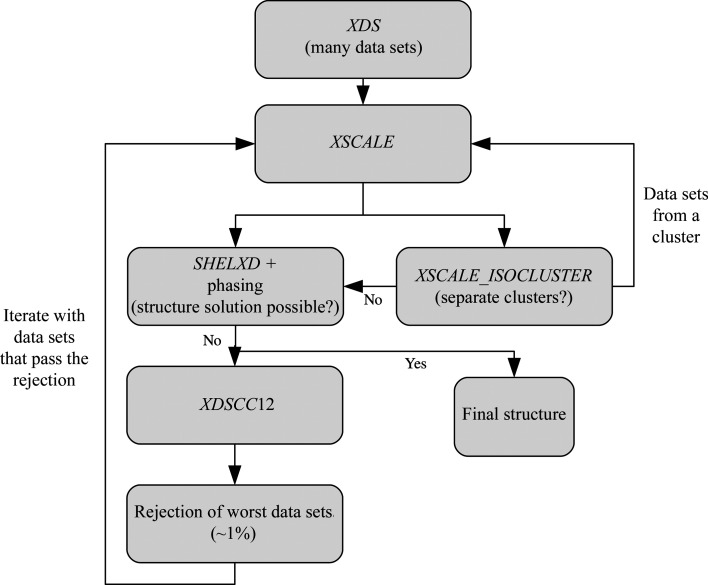
Flow chart of the main processing steps.

A flow chart of the main processing steps is shown in Fig. 1 ▸.

## 3. Results   

### 3.1. *XSCALE_ISOCLUSTER*   

**Figure 2:**
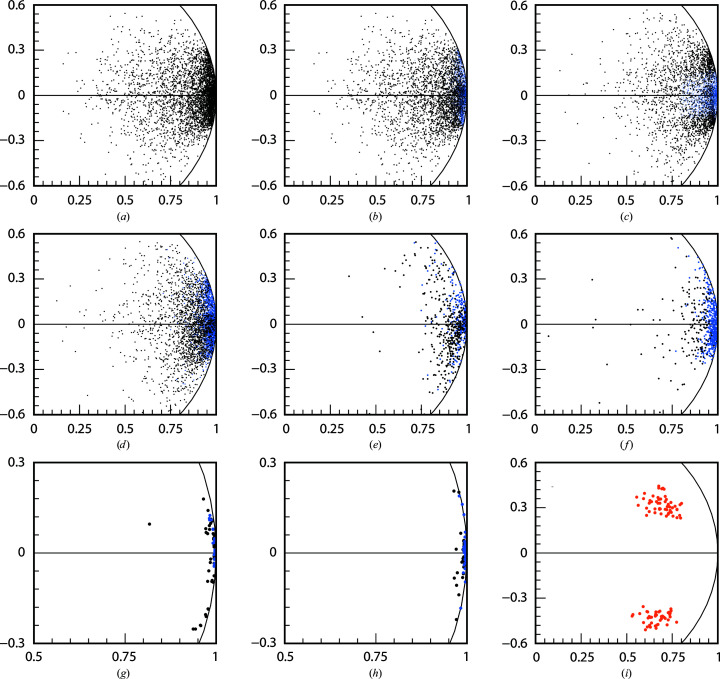
Analysis of the data sets with *XSCALE_ISOCLUSTER*. The *x* and *y* axes represent a two-dimensional scaling analysis (equation 1) and a section of the unit circle is shown: (*a*) PepT, all 4528 data sets; (*b*) PepT, selection (blue) of the 1595 data sets suggested by Huang *et al.* (2018 ▸) for structure solution; (*c*) PepT, selection (blue) of 2162 data sets with length > 0.8 and |angle| ≤ 10°. (*d*)–(*h*) Rejected data sets (black) at iteration 40 of iterative application of *XDSCC*12 and remaining data sets (blue) for (*d*) PepT, (*e*) BacA, (*f*) LspA, (*g*) NS1 and (*h*) modified 1g1c; (*i*) analysis before re-indexing is shown in orange for modified 1g1c.

For PepT, 4528 data sets were analyzed. *XSCALE_ISO­CLUSTER* showed no clear separation of data sets or clusters (Fig. 2 ▸ *a*). Therefore, we tried several subsets with different cutoffs of length and angle (within a cone relative to the centre of gravity) in the ranges 0.5–0.95 and 5–20°, respectively (for example, Fig. 2 ▸ *c* shows length 0.8 and angle ±10°].

Selecting vectors with length > 0.8 resulted in 4068 data sets enabling structure solution, but resulted in a lower CFOM (39.8) than the 1595 data sets selected by Huang *et al.* (2018 ▸) (CFOM = 43.6; Fig. 2 ▸ *b*). At a higher length threshold (0.9; 3022 data sets) the CFOM rose to 46.0. In contrast, subset generation dependent on the angle alone did not enable structure solution. Combined selection of length and angle also enabled structure solution, but the results were not sub­stantially improved relative to selection based on length alone.

For BacA, selections based on length alone were attempted but did not lead to structure solution. For LspA, selections based on length were attempted and led to structure solution. This was expected, as the LspA structure could already be solved without any rejections, and further improvement of the signal inevitably resulted in structure solution as long as the completeness was maintained, which was the case. No attempts to select based on length were made for NS1 and modified 1g1c since the structures could be solved without selection.

A visualization of the analysis of the data sets of the three SSX projects with *XSCALE_ISOCLUSTER* after the application of *XDSCC*12 (see Sections 3.2–3.5) is shown in Figs. 2 ▸(*d*), 2 ▸(*e*) and 2 ▸(*f*). Rejected data sets after an arbitrary number of iterations (40 in each project) mainly represent high random error and high systematic error.

Visualization in the unit circle of the 62 complete experimental data sets of NS1 in Fig. 2 ▸(*g*) shows that mainly data sets with high random and systematic error are rejected by the ΔCC_1/2_-based iterations. The 100 data sets of modified 1g1c analyzed using *XSCALE_ISO­CLUSTER* are represented in Figs. 2 ▸(*h*) and 2 ▸(*i*). Before resolving the indexing ambiguity, these data sets fall into two clusters with a distinct 90° separation, as shown in Fig. 2 ▸(*i*). After re-indexing, they form a single cluster (Fig. 2 ▸ *h*), and ΔCC_1/2_-based iterations reject data sets without any obvious selection pattern. The arrangement of vectors is extended perpendicular to the radial direction of low-dimensional space; this indicates systematic differences which cannot be compensated by scaling, for example radiation damage or differences in unit-cell parameters.

The difference between data sets rejected based on ΔCC_1/2_ and the remaining data sets is not apparent in any of the *XSCALE_ISOCLUSTER* analyses, as data sets with low random and low systematic error are also sometimes rejected.

### 3.2. *XDSCC*12: common findings for the partial experimental SSX data sets   

The three projects with partial experimental SSX data sets can be classified as a challenging project (BacA), where structure solution without manual model building is barely possible, a project where structure solution is only possible after rejection of the worst data sets (PepT), and a less challenging project where structure solution is already possible with all data sets but further improvement can be made through rejection of the worst data sets (LspA).

**Figure 3:**
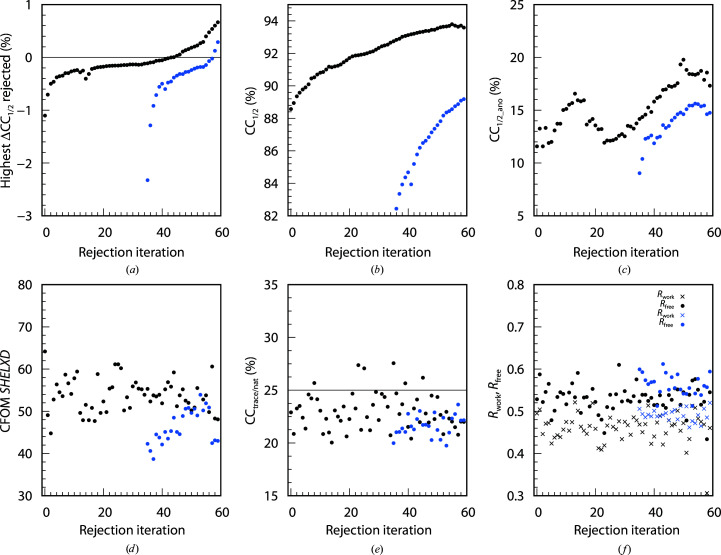
60 rejection iterations of BacA (724 data sets): ten data sets are rejected per iteration. *XDSCC*12 analysis performed with all data sets is shown in black and that performed with a random half is shown in blue. (*a*) Highest ΔCC_1/2,*i*_ of the rejected data sets, (*b*) CC_1/2_, (*c*) CC_1/2_ano_, (*d*) the best *SHELXD* CFOM solutions, (*e*) CC_trace/nat_ from *SHELXE* and (*f*) *R*_work_ (crosses) and *R*_free_ (circles) from *REFMAC* in the *CRANK*2 pipeline.

**Figure 4:**
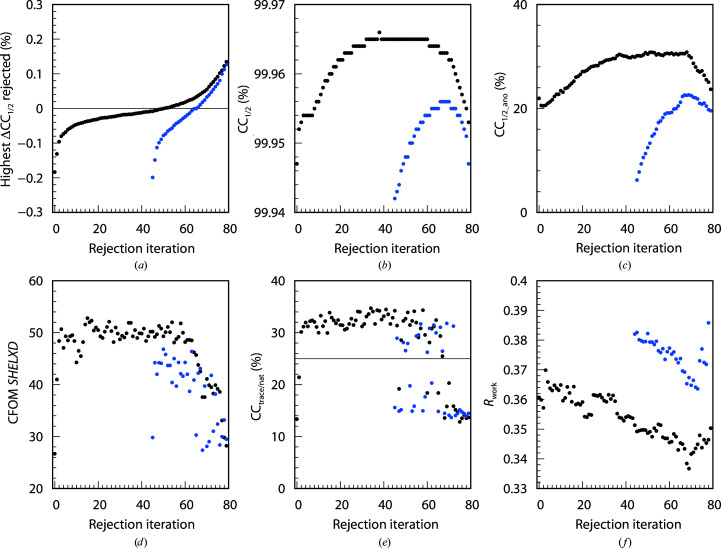
80 rejection iterations of PepT (4528 data sets): 50 data sets are rejected per iteration. *XDSCC*12 analysis performed with all data sets is shown in black and that performed with a random half is shown in blue. (*a*) Highest ΔCC_1/2,*i*_ of the rejected data sets, (*b*) CC_1/2_, (*c*) CC_1/2_ano_, (*d*) the best *SHELXD* CFOM solutions, (*e*) CC_trace/nat_ from *SHELXE* and (*f*) *R*_work_ from *phenix.refine*.

**Figure 5:**
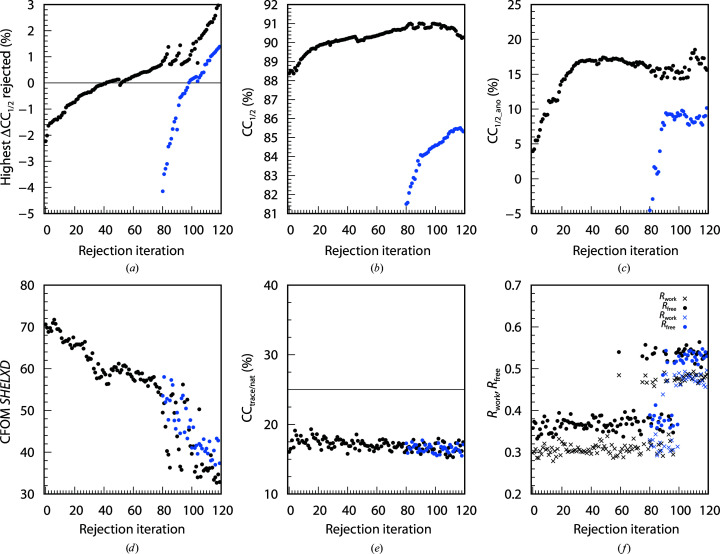
120 rejection iterations of LspA (614 data sets): four data sets are rejected per iteration. *XDSCC*12 analysis performed with all data sets is shown in black and that performed with a random half is shown in blue. (*a*) Highest ΔCC_1/2,*i*_ of the rejected data sets, (*b*) CC_1/2_, (*c*) CC_1/2_ano_, (*d*) the best *SHELXD* CFOM solutions, (*e*) CC_trace/nat_ from *SHELXE* and (*f*) *R*_work_ (crosses) and *R*_free_ (circles) from *REFMAC* in the *CRANK*2 pipeline.

The 742, 4528 and 614 data sets of the BacA (Fig. 3 ▸), PepT (Fig. 4 ▸) and LspA (Fig. 5 ▸) projects, respectively, were analysed with *XDSCC*12. Application of the rejection procedure in order to optimize CC_1/2_ was conducted as described above. ΔCC_1/2,*i*_ was calculated by *XDSCC*12 for every data set. Rejection of the worst ten, 50 and four data sets, respectively, corresponding to about 1% of all data sets, was performed iteratively. An attempt to solve the structure with *SHELXC*/*D*/*E* or *CRANK*2 was made at each rejection cycle. The whole procedure was performed starting with all data sets (black curves in Figs. 3 ▸, 4 ▸ and 5 ▸) and also starting with a randomly chosen half of the data (blue curves). Quantities from half of the data are offset in Figs. 3 ▸, 4 ▸ and 5 ▸ by 35, 45 and 80 iterations, respectively, since in these iterations the number of randomly omitted data sets roughly corresponds to the numbers in the rejection rounds with all of the data sets. In these projects, the multiplicity was so high that the rejection of data sets did not compromise the completeness of the resulting merged data within the range of rejection iterations shown in Figs. 3 ▸, 4 ▸ and 5 ▸.

A total of 60, 80 and 120 iterations, respectively, were calculated in order to investigate the asymptotic behaviour of ΔCC_1/2_, CC_1/2_, CC_1/2_ano_, CFOM, CC_trace/nat_ and refinement *R* values of *CRANK*2 solutions.

Figs. 3 ▸(*a*), 4 ▸(*a*) and 5 ▸(*a*) show the highest ΔCC_1/2,*i*_ values of all data sets rejected in each iteration. The first iterations show strongly negative values; after iterations 50, 50 and 60, respectively, positive data sets are rejected and subsequently strongly positive data sets. The ΔCC_1/2,*i*_ values of half of the data also show strong negative values at the beginning; data sets with positive ΔCC_1/2,*i*_ values are rejected in the last iterations.

We observe that in parallel with the optimization of CC_1/2_ (Figs. 3 ▸ *b*, 4 ▸ *b* and 5 ▸ *b*), CC_1/2_ano_ on average increases during the rejection iterations both for all data sets and half of the data, but decreases slightly for the last iterations (Figs. 3 ▸ *c*, 4 ▸ *c* and 5 ▸ *c*) when data sets with positive ΔCC_1/2,*i*_ values are rejected. Quantitatively, the correlation between CC_1/2_ and CC_1/2_ano_ is 0.66 for BacA, 0.92 for PepT and 0.79 for LspA.

The CFOM (CFOM = CC_weak_ + CC_all_) of the best *SHELXD* solution per 25 000 attempts is depicted in Figs. 3 ▸(*d*), 4 ▸(*d*) and 5 ▸(*d*). It shows the highest values after a few rounds of rejections at the beginning, decreasing with following iterations for both all data sets and half of the data. CFOM values for half of the data are in general lower than the values for all the data. The *SHELXE* CC_trace/nat_ values (the best obtained in 25 autotracing cycles) are shown in Figs. 3( ▸ *e*), 4 ▸(*e*) and 5 ▸(*e*), indicating no successful structure solution for BacA and LspA and indicating success for PepT.

In general it is found that a decrease in CC_1/2_ (Figs. 3 ▸ *b*, 4 ▸ *b* and 5 ▸ *b*), CC_1/2_ano_ (Figs. 3 ▸ *c*, 4 ▸ *c* and 5 ▸ *c*), worse *SHELXD* solutions (Figs. 3 ▸ *d*, 4 ▸ *d* and 5 ▸ *d*), insufficient *SHELXE* results (Figs. 3 ▸ *e*, 4 ▸ *e* and 5 ▸ *e*) and an increase in *R* values (Figs. 3 ▸ *f*, 4 ▸ *f* and 5 ▸ *f*) arise from the rejection of data sets with positive ΔCC_1/2,*i*_ values (Figs. 3 ▸ *a*, 4 ▸ *a* and 5 ▸ *a*).

Application of the iterative rejection procedure to all data sets enables a noticeable improvement in the final merged data, which simplifies structure solution compared with the previous work (Huang *et al.*, 2018 ▸). Similar improvements are seen in a random selection of half of the available data sets.

### 3.3. *XDSCC*12: individual findings for BacA   

The most challenging project (BacA) shows a varying, relatively low CFOM for the best *SHELXD* solution of between 50 and 60 (Fig. 3 ▸ *d*). The *SHELXD* solutions are improved after rejecting the worst data sets in both all-data and half-data tests. Compared with previous work (Huang *et al.*, 2018 ▸) the substructure determination is easier, whereas structure solution is still difficult: the best CC_all/weak_ (CFOM) from *SHELXD* for BacA with 360 data sets selected by Huang *et al.* (2018 ▸) are 29.4/17.1 (46.5) and the best CC_all/weak_ (CFOM) from this study are 38.7/25.5 (64.2) with all 724 data sets.

The CC_trace/nat_ values are mostly below 25%, failing to indicate structure solution both for all and half of the data (Fig. 3 ▸ *e*). However, an additional diagnostic, the weighted mean phase error (wMPE) calculated by *SHELXE* with the PDB reference model 6fmt, reveals a wMPE of ∼70°. This indicates a basically correct but incomplete solution for almost all iterations. Consistent with this, *R*_free_ values of the order of 45% result from a few iterations of the *CRANK*2 pipeline (Fig. 3 ▸ *f*) with all data sets, also indicating successful structure solution.

In contrast, CC_trace/nat_ of half of the data is below 25% for all iterations and the wMPE is mostly at ∼90°, which indicates failure of structure solution. Consistently, the *R* values in this case do not indicate structure solution.

### 3.4. *XDSCC*12: individual findings for PepT   

The PepT project shows low CFOM values of the best *SHELXD* solution for the first two iterations in Fig. 4 ▸(*d*). Consistent with this, the CC_trace/nat_ values indicate no solution in the first two iterations in Fig. 4 ▸(*e*). The same is true for half of the data; solutions can be found only after the first rejection iteration and for a few of the following iterations.

Compared with the original publication, the structure solution is much easier for any rejection round between 3 and 65: the best CC_all/weak_ (CFOM) for PepT with 1595 data sets selected by Huang *et al.* (2018 ▸) are 31.0/12.6 (43.6), whereas the best CC_all/weak_ (CFOM) found in this study are 34.0/18.8 (52.8) with 3778 data sets.

Application of the iterative rejection procedure results in better data quality, improved *SHELXD* solutions and enables structure solution. This SSX case study with PepT shows that a few iterations which reject the worst data sets make the difference in structure solution for both all and half of the data.

*R*_work_ in the highest resolution shell (2196 reflections) from the refinement of the merged data of each iteration with the shaken PDB model 4xnj is depicted in Fig. 4 ▸(*f*). These *R* values decrease up to iteration ∼65, indicating an improvement of data quality in high-resolution shells, and continuously increase afterwards both for all and half of the data. *R*_free_ on average decreases in parallel (data not shown), but the variation is much higher since the number of test reflections is only 107.

### 3.5. *XDSCC*12: individual findings for LspA   

The least challenging project, LspA, has CC_trace/nat_ lower than 20% (Fig. 5 ▸ *e*), which is less than expected for successful structure solution. This is found when using all of the data sets and for a random selection consisting of half of the data sets. However, *R*_free_ from the final refinement step of the *CRANK2* pipeline (Fig. 5 ▸ *f*) using the previously found *SHELXD* solutions clearly indicates successful structure solution up to rejection iteration 95 starting with all of the data sets. When starting the rejection iterations with half of the 614 data sets, solutions can be found only for the first 20 iterations.

Compared with the original publication the structure solution is eased: the best CC_all/weak_ (CFOM) for LspA with 497 data sets selected by Huang *et al.* (2018 ▸) are 41.5/16.5 (58.0), whereas the best CC_all/weak_ (CFOM) from this study are 45.7/26.0 (71.7) with 590 data sets.

Application of the iterative rejection procedure to all data sets thus results in significantly better data quality and enables structure solution without rejection steps, even with only half of the data.

### 3.6. *XDSCC*12: complete experimental data sets for NS1   

**Figure 6:**
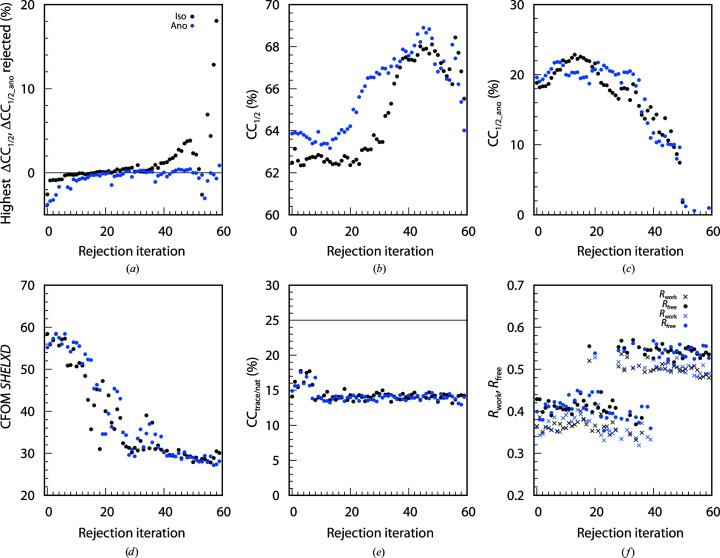
60 rejection iterations of NS1 (62 data sets). *XDSCC*12 analysis performed with all data sets based on ΔCC_1/2_ (black) and based on ΔCC_1/2_ano_ (blue). (*a*) Highest ΔCC_1/2,*i*_ and ΔCC_1/2_ano,*i*_ of the rejected data sets, (*b*) CC_1/2_, (*c*) CC_1/2_ano_, (*d*) the best *SHELXD* CFOM solutions, (*e*) CC_trace/nat_ from *SHELXE* and (*f*) *R*_work_ (crosses) and *R*_free_ (circles) from the *CRANK*2 pipeline.

The rejection procedure that optimizes CC_1/2_ was applied to 62 complete data sets obtained with *XDS* from raw data (derived from 28 crystals; Akey *et al.*, 2014 ▸) and serving as an example of multi-data-set crystallography with complete data sets (Fig. 6 ▸). Optimization based on both ΔCC_1/2,*i*_ (blue curves) or ΔCC_1/2_ano,*i*_ (black curves) was performed, as the data sets provide sufficient reflections to calculate significant ΔCC_1/2_ano,*i*_ values. In each iteration, the worst data set was rejected. 60 iterations were calculated in total, although the structure could already be solved without rejection (Fig. 6 ▸ *f*). Again, this was performed to investigate the behaviour of ΔCC_1/2,*i*_, CC_1/2_, CC_1/2_ano,*i*_ and *SHELXD*/*E* solutions in further iterations.

Fig. 6 ▸(*a*) shows the highest ΔCC_1/2,*i*_ and ΔCC_1/2_ano,*i*_ of all data sets rejected in each iteration. Both quantities increase continuously, and data sets with positive ΔCC_1/2,*i*_ are rejected from iteration 20 onwards, consistent with the decline of CC_1/2_ano,*i*_ (Fig. 6 ▸ *c*). We observe an increase of CC_1/2_ (Fig. 6 ▸ *b*) and CC_1/2_ano_ (Fig. 6 ▸ *c*) for optimization based on either ΔCC_1/2,*i*_ or ΔCC_1/2_ano,*i*_. CC_1/2_ decreases from iteration 45 onwards, whereas CC_1/2_ano_ starts to decrease from iteration 20.

The CFOM of the best *SHELXD* solution per 25 000 attempts is depicted in Fig. 6 ▸(*d*). For both selection strategies, the best CFOM decreases with increasing iteration. The CC_trace/nat_ values are shown in Fig. 6 ▸(*e*). They are lower than 20%, thus not indicating structure solution. However, using *CRANK*2 the structure can be solved without rejection from the first iteration onwards for the next ∼40 iterations for either ΔCC_1/2_ or ΔCC_1/2_ano_ optimization, as shown in Fig. 6 ▸(*f*) representing *R*_free_ and *R*_work_ from the *CRANK*2 pipeline.

No significant difference between ΔCC_1/2_ and ΔCC_1/2_ano_ optimization can be observed; both serve well as optimization targets. In contrast to the findings of the original publication (Akey *et al.*, 2014 ▸), the structure was solved over a wide range of data-set numbers and even without rejections. We attribute this to improvement in all procedures contributing to structure solution.

### 3.7. *XDSCC*12: simulated SSX data sets   

The challenge prepared by Holton (2019 ▸) was threefold: firstly to resolve the indexing ambiguity arising from two axes of the same length in an orthorhombic space group, secondly to cope with strong radiation damage in scaling, and thirdly to find the minimal number of data sets for structure solution using the (simulated) anomalous signal of selenomethionine

The first challenge was met by using *XSCALE_ISOCLUSTER* to identify the two groups of data sets which differ in their indexing mode (Fig. 2 ▸ *h*). Based on this result, data sets of one of the groups were re-indexed in *XSCALE* and merged with the data sets of the other group. The second challenge was tackled by increasing (to 3, from the default of 1) the number of scale factors used for the DECAY (*i.e.* radiation damage) scaling in *XSCALE*. The solutions of these challenges were obtained in previous work but not formally published (XDSwiki; https://strucbio.biologie.uni-konstanz.de/xdswiki/index.php/SSX).

**Figure 7:**
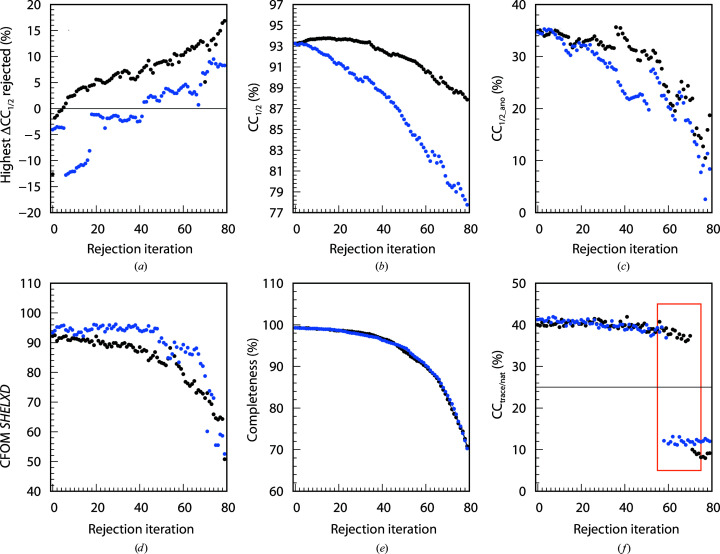
80 rejection iterations of modified 1g1c (100 data sets): one data set is rejected per iteration. *XDSCC*12 analysis performed with all data sets based on ΔCC_1/2_ is shown in black. Random rejection is shown in blue. (*a*) Highest ΔCC_1/2,*i*_ of the rejected data sets, (*b*) CC_1/2_, (*c*) CC_1/2_ano_, (*d*) the best *SHELXD* CFOM solutions, (*e*) completeness and (*f*) CC_trace/nat_ from *SHELXE*. The range of iterations where random and ΔCC_1/2_-based rejections differ is highlighted by an orange rectangle.

The goal of this study was mainly to meet the third challenge. To this end, the rejection of the worst data set in order to optimize CC_1/2_ was performed 80 times for the 100 data sets (Fig. 7 ▸, black curves). As a control, the sequential omission of one data set per iteration, as performed by Holton (2019 ▸), which is equivalent to random rejection, was performed 80 times (Fig. 7 ▸, blue curves).

Fig. 7 ▸(*a*) shows the highest ΔCC_1/2,*i*_ value of all data sets rejected in each iteration. It increases steadily, and data sets with positive ΔCC_1/2,*i*_ start to be rejected after a few iterations. In contrast to this, the random rejection shows varying ΔCC_1/2,*i*_ values of the rejected data set, as expected.

In Figs. 7 ▸(*b*) and 8 ▸(*c*) for the ΔCC_1/2_-based optimization we observe a decrease in CC_1/2_ and CC_1/2_ano_, respectively, for almost all iterations after the first iteration. CC_1/2_ and CC_1/2_ano_ for random rejection are in general lower, but show the same behaviour.

The CFOM of the best *SHELXD* solution per 25 000 attempts is depicted in Fig. 7 ▸(*d*). For both random and ΔCC_1/2_-based rejection, the best CFOM decreases with increasing iteration number. The best CFOM values based on random rejection are in general higher than the CFOM values of the rejection based on ΔCC_1/2_.

The completeness of the merged data set for each iteration is shown in Fig. 7 ▸(*e*). For both rejection algorithms the completeness decreases with increasing iterations.

The CC_trace/nat_ values are shown in Fig. 7 ▸(*f*). The structure can be solved in all iterations down to a minimum of 30 data sets if data sets are rejected based on ΔCC_1/2_. We believe that the lack of completeness (about 80% in all resolution ranges when only 30 data sets remain) becomes the limiting factor for successful structure solution.

In comparison, the structure is solved for every iteration down to a minimum of 42 data sets (as found by Holton, 2019 ▸) if data sets are randomly rejected.

### 3.8. *XDSCC*12: technical aspects of the scaling method and ΔCC_1/2_ calculation   

For the PepT project only, we assessed the importance of individual elements of the rejection iterations as follows.

(i) By omitting the reset of the variance model.
(ii) By using frequency weights (3) in *XDSCC*12 instead of reliability weights (4).
(iii) By using no Fisher transformation in *XDSCC*12, *i.e.* using (5) instead of (6).
(iv) By random rejection instead of ΔCC_1/2,*i*_-based rejection.

**Figure 8:**
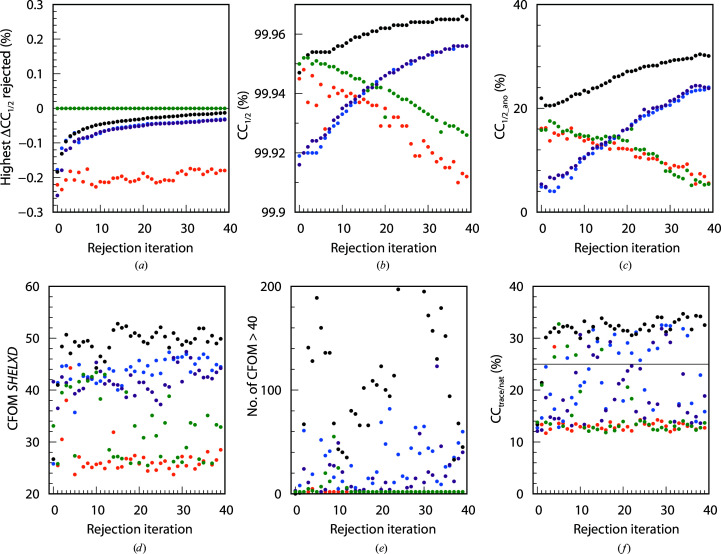
40 rejection iterations of PepT (4528 data sets): 50 data sets are rejected per iteration. *XDSCC*12 analysis performed with all data sets based on (4) (weighted ΔCC_1/2,*i*_) is shown in black, that with unweighted ΔCC_1/2,*i*_ (3) in blue, that without Fisher transformation in green and that without resetting the error model in dark violet. Random rejection is shown in orange. (*a*) Highest ΔCC_1/2,*i*_ of the rejected data sets, (*b*) CC_1/2_, (*c*) CC_1/2_ano_, (*d*) the best *SHELXD* CFOM solutions, (*e*) the number of *SHELXD* CFOM solutions > 40.0 in 25 000 attempts and (*f*) CC_trace/nat_ from *SHELXE*.

40 rejection iterations were used in each case. Fig. 8 ▸(*a*) shows the highest ΔCC_1/2,*i*_ of all rejected data sets, Fig. 8 ▸(*b*) shows CC_1/2_, Fig. 8 ▸(*c*) shows CC_1/2_ano_, Fig. 8 ▸(*d*) shows the best CFOM solutions, Fig. 8 ▸(*e*) shows the number of ‘high’ *SHELXD* solutions per 25 000 attempts and Fig. 8 ▸(*f*) shows CC_trace/nat_ for all five alternatives.

We find that random rejection performs worst, as expected. Rejection based on ΔCC_1/2,*i*_ without Fisher transformation enables structure solution for only six out of 40 rejection iterations. CC_1/2_ and CC_1/2_ano_ decrease constantly, the best CFOM values are low and almost no ‘high’ *SHELXD* solutions are found. The highest ΔCC_1/2,*i*_ values (Fig. 8 ▸ *a*) of all rejected data sets are slightly below zero for all iterations.

Use of *XDSCC*12 without reliability weights or without resetting the variance model shows increasing CC_1/2_ and CC_1/2_ano_, but enables structure solution for only 25 and 17 out of 40 rejection iterations, respectively. The best CFOM solutions are higher than for random rejection, and more ‘high’ *SHELXD* solutions are found.

As shown in Fig. 8 ▸, rejection based on ΔCC_1/2,*i*_ with reliability weights in combination with upstream resetting of the variance model and Fisher transformation, *i.e.* the procedure combining the methodological improvements that we suggest in this study, improves the anomalous signal (CC_1/2_ano_) significantly (Fig. 8 ▸ *c*), has the best CFOM solutions and the highest number of ‘high’ *SHELXD* solutions (Figs. 8 ▸ *d* and 8 ▸ *e*), and enables structure solution in all except for the first two iterations.

## 4. Discussion   

The paradigm of multi-data-set scaling and merging is that averaging reduces random errors in the merged intensities, according to the laws of error propagation. However, this assumes that the intensity differences of different data sets with respect to the unknown ‘true’ intensities are unrelated, which does not hold in the case of non-isomorphism. If the data sets have systematic differences, merging introduces systematic errors that are not necessarily reduced by averaging. Without non-isomorphism, the accuracy of the merged data is identical to their precision, for which a number of crystallographic indicators exist. However, in the presence of systematic differences (the crystallographic term for which is ‘non-isomorphism’), the accuracy of the merged data is worse than their precision by an amount that is difficult to quantify, but which can be large enough to prevent structure solution.

Our finding in this work is that non-isomorphous data sets can be identified by the computational tools *XSCALE_ISOCLUSTER* and *XDSCC*12 and that their rejection results in merged and averaged data that are better suited for experimental phasing, structure solution and refinement.

*XSCALE_ISOCLUSTER* was used in all projects described here to find out whether there are distinct subgroups in the data sets. It was our hope and expectation that subgroups may represent distinct and different conformations or packings of the molecules, and that scaling and merging within each subgroup may yield opportunities for insight into the bio­logically relevant conformations that are accessible by the crystallized proteins.

However, except for the modified 1g1c project, where the use of *XSCALE_ISOCLUSTER* was instrumental, we did not find obvious subgroups in any of the projects that would have enabled us to analyze possible alternative structures. Removal of outliers based on direction in the low-dimensional representation of the data sets was tried, but we found no simple algorithm to perform this sensibly. One reason for this failure to identify subgroups is the fact that partial data sets on average have only a low number of reflections in common. This results in large standard errors of the correlation coefficients calculated from the common reflections, and gives rise to deviations of the vectors from their ideal angles, thus diminishing the signal that could be used to identify subgroups. Even more importantly, the set of common reflections is different for each pair of data sets if these are partial, which leads to correlation coefficients CC_*i*,*j*_ that are not strictly comparable. This is only partially compensated by the fact that the low-dimensional vectors are highly overdetermined if many data sets are available. Another reason may be that our choice of projects is biased towards those that were previously solved using less advanced methods, possibly because no such subgroups existed.

On the other hand, the modified 1g1c project demonstrates that *XSCALE_ISOCLUSTER* is a valuable tool to identify major systematic differences in SSX data sets. A distinct separation of data sets in terms of direction is a reliable indicator, and allows either rejection or different treatment (for example re-indexing) of the separated data sets. Clusters of data sets can be selected according to random properties (vector length) and systematic properties (direction) and processed separately, as was performed to resolve the indexing ambiguity of the simulated SSX data. Therefore, we suggest that *XSCALE_ISOCLUSTER* should be applied to SSX data to detect distinct clusters or indexing issues before outlier removal using *XDSCC*12 is initiated. Future work will investigate algorithmic improvements through Fisher transformation of correlation coefficients and scalar products in (1) and weighting of its terms with the number of common reflections.

*XDSCC*12 implements a target function that allows the large number of possible combinations of data sets to be conquered by a greedy algorithm, *i.e.* an efficient procedure that ranks the data sets by their contribution towards the CC_1/2_ of the final, merged data set. By doing so, *XDSCC*12 enables the reliable rejection of outlier data sets which, after rescaling the remaining data sets, first and foremost improves the precision of merged data to the point where difficult projects can be solved. Our results confirm that data sets with negative ΔCC_1/2,*i*_ are non-isomorphous relative to the bulk of the other data sets and that their exclusion improves the overall level of isomorphism. Rejection and subsequent scaling of data sets should be iterated at most until the rejected data sets show a positive ΔCC_1/2,*i*_, since further rejection iterations noticeably deteriorate the signal and ultimately prevent downstream structure solution.

The type or nature of non-isomorphism that is present in the rejected data sets cannot in general be derived from ΔCC_1/2_, and a significant correlation of ΔCC_1/2_ with unit-cell differences from the average was not found in the projects that we investigated (data not shown). For the simulated modified 1g1c project, we found a rejection preference for smaller (<100 µm^3^) crystals, but some large crystals were also rejected. To further assess the possibility that an alternative and simpler procedure could outperform our ΔCC_1/2_-based scaling/rejection procedure for modified 1g1c, we ran rejection iterations based on crystal size only, but found that this was about as successful as random rejection.

The statistics for all projects (Figs. 3 ▸, 4 ▸, 5 ▸, 6 ▸, 7 ▸ and 8 ▸) are consistent with the interpretation of ΔCC_1/2_ as a non-isomorphism indicator since they initially show an increase in CC_1/2_ and CC_1/2_ano_ when rejecting data sets with negative ΔCC_1/2_. As expected, this improves substructure determination, as shown by significant increases in the CFOM values. Additionally, a promising aspect of data selection by ΔCC_1/2_ is the improvement of a model by refinement with the selected merged data set, as shown in the PepT case, where we monitored *R*_work_ for the highest resolution shell. Consistently, in all projects both CC_1/2_ and CC_1/2_ano_ deteriorate upon the rejection of data sets with positive ΔCC_1/2._

Our results thus validate the choice of CC_1/2_ as a target function, and in particular an approach that scales and scores each data set in the context of all other data sets. Our method avoids arbitrary cutoffs, but instead uses ΔCC_1/2_ = 0 as the natural threshold between data sets that are isomorphous and those that are not.

Would it be possible to devise an alternative but analogous procedure attempting to optimize, for example, the mean *I*/σ, *R*_meas_ or completeness as a target function? In the case of optimization of the mean *I*/σ, once the data sets are scaled the *I*/σ of each unique reflection increases on average with every additional observation (*I*_*i*_, σ_*i*_). This is because the intensity *I* on average does not change, since scaling results in the intensities of all observations of a unique reflection being approximately equal, but σ decreases monotonically with every additional observation according to
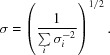



If *I*/σ of each unique reflection increases on average, so does the mean *I*/σ. This thought experiment reveals that every data set would display a positive Δ*I*/σ; data sets could still be ranked in such a procedure, but ranking on Δ*I*/σ would just reproduce the ranking of the *I*/σ values, independent of any possible non-isomorphism. This property would defeat the purpose of the optimization. In addition, an explicit Δ*I*/σ optimization appears to be unsuitable as although it is known that there is a practical difficulty in estimating accurate σ_*i*_ values in a data-processing package, the *I*/σ calculation explicitly assigns an important role to the σ_*i*_ values.

Choosing *R*_meas_ as a component of a target function in our view would not necessarily improve the final result since *R*_meas_ indicates the precision of the unmerged data (individual observations) rather than that of the merged data, and thus favours strong data sets regardless of their level of non-isomorphism. However, in ‘easy’ cases optimizing *R*_meas_ may lead to structure solution, as may happen with any other method that just rejects weak data.

Completeness does not appear to be required as an explicit component of a target function, as optimization of CC_1/2_ alone automatically favours high completeness for a given number of data sets, as is shown by the results for simulated 1g1c.

Most importantly, and at the same time somewhat unexpectedly and encouragingly to us, the improvement of the anomalous signal (CC_1/2_ano_) and the success of substructure determination run parallel to the improvement of the isomorphous signal (CC_1/2_), even if just the latter is explicitly optimized by rejecting data sets based on ΔCC_1/2_. The anomalous signal, which owing to its low magnitude can easily be swamped by noise, benefits from the exclusion of data sets with negative ΔCC_1/2_, leading to high correlation (0.66, 0.92 and 0.79 for BacA, PepT and LspA, respectively) between CC_1/2_ano_ and CC_1/2_ for the three experimental SSX projects that we investigated. This demonstrates that our rejection procedure improves not only the precision of the merged data, but also, much more importantly, their accuracy.

When implementing and testing *XDSCC*12, we identified a number of technical aspects that each substantially improve the target function on their own, and even more so when taken together.

(i) The postponement of the scaling and estimation of the error model from *XDS* (using SNRC=50 or resetting the error model) to *XSCALE* ensures consistent variances of the observations, regardless of the number of symmetry-related observations within a data set. This results in better anomalous signal not only for highly partial data sets, where the error model cannot be reliably determined without reference to the other data sets, but also in cases with almost complete data sets (data not shown). We believe that the postponed global adjustment of the error model, which typically increases the σ of the strong reflections, results in higher weights for the low-resolution reflections at the start of the scaling iterations in *XSCALE*, and as a consequence yields lower systematic differences for these, which enhances the anomalous signal.
(ii) The inclusion of reliability weights (4) in the calculation of CC_1/2_ is essential to obtain correct CC_1/2_ values and the respective differences, as the reliability weights reduce the bias in the weighted estimator for σ_∊_^2^. This procedure also improves CC_1/2_ano_ significantly in all cases tested in this study.
(iii) Fisher transformation of the ΔCC_1/2_ values is performed to obtain meaningful differences independent of the magnitude of the CC_1/2_ values involved. We believe that this is particularly important in the case of significantly anisotropic data.

Our results show that taken together these measures improve, relative to variations of the procedure, the merged data for substructure solution using the anomalous signal and for model building and refinement using the isomorphous signal.

Additional work will be required to determine whether further improvement of the merged data can be obtained by a more fine-grained rejection based on resolution shells of data sets, instead of the rejection of complete data sets, by using the ΔCC_1/2,*i*_ values for each resolution range.

Besides the application of *XDSCC*12 to multi-data-set projects, as shown in this study, the program can also be used for frame ranges (for example encompassing 1° of rotation) of single (complete) data sets. This helps to detect frame ranges that deteriorate the CC_1/2_ of the data set, for example owing to radiation damage, owing to the crystal moving out of the X-ray beam during rotation or owing to reflections from a second crystal interfering with integration of the main crystal. This function of the program is documented in XDSwiki (https://strucbio.biologie.uni-konstanz.de/xdswiki/index.php/Xdscc12) and is used to produce a ΔCC_1/2_ plot in *XDSGUI* (Brehm & Diederichs, to be published). Moreover, we also consider the application of *XDSCC*12 to SFX data or data with still images in general. This should also enable the optimization of merged data from clusters of isomorphous SFX shots after their identification with *XSCALE_ISOCLUSTER* (for an example with data from photosystem I, see Diederichs, 2017 ▸). For such data, our methods will greatly benefit from the progress made in partiality estimation.

SSX has emerged as a viable tool for macromolecular crystallography, and enables structure determination from weakly diffracting microcrystals that were previously intractable. To ensure its successful applications at macromolecular crystallography beamlines, robust data-set selection methods become essential. Our methods offer a fast and deterministic approach and can readily be incorporated into beamline pipelines. As demonstrated in the three SSX test cases, structure solutions can be found with half of the data previously required. Therefore, not only can sample consumption be significantly reduced, but the synchrotron beamtime can also be used more efficiently. We expect that this work will help in making SSX a routine structure-determination method for structural biologists.

## Figures and Tables

**Table 1:** Statistics of data sets used in this study

	PepT (S)	BacA (Hg)	LspA (Se/S†)	NS1 (S)	Modified 1g1c (Se)
No. of crystals processed	4528	742	614	28	100
No. of data sets merged in the original publication	1595	360	497	18	100
Resolution *d*_min_ (Å)	2.7	3.0	3.0	2.9	1.8
Space group	*C*222_1_	*C*222	*C*2	*P*321	*P*2_1_2_1_2_1_
Fractional solvent content	0.55	0.60	0.65	0.65	0.53
Multiplicity	1002.8	126.7	27.2	114.8	5.1
PDB code	6fmy	6fmt	6fms	4tpl	Derived from 1g1c
Average rotation range per data set (°)	10–20	10–20	10–20	90	3
Type and No. of anomalous scatterers for substructure search	12 S	2 Hg	12 Se	30 S	4 Se
Resolution cutoff for substructure search (Å)	3.5	3.3	4.2	4.2	3.5
Best CC_all_/CC_weak_ from publication (%)	31.0/12.6	29.4/17.1	41.5/16.5	Not available	Not available
Structure-solution software	*SHELXC*/*D*/*E*	*SHELXC*/*D* + *CRANK*2	*SHELXC*/*D* + *CRANK*2	*SHELXC*/*D* + *CRANK*2	*SHELXC*/*D*/*E*

† The crystals contain a mixture of selenomethionine-labelled and native protein.
